# A community- based hepatitis B linkage-to-care program: a case study on Asian Americans chronically infected with hepatitis B virus

**DOI:** 10.1186/s41124-016-0006-8

**Published:** 2016-05-05

**Authors:** Chul S. Hyun, William R. Ventura, Soon S. Kim, Soyoung Yoon, Seulgi Lee

**Affiliations:** 1 Holy Name Medical Center, Teaneck, NJ USA; 2 Center for Viral Hepatitis, 35 Van Nostrand Avenue, Englewood, NJ 07631 USA; 3 St. Joseph’s Regional Medical Center, Patterson, NJ USA; 4 Korean Community Services, Queens, NY USA

**Keywords:** Health disparity, Hepatitis B, Linkage to care, Patient navigator, Community program

## Abstract

**Background:**

Hepatitis B is an important disease of ethnic disparity which affects Asian Americans and other minority populations disproportionately. Despite the high prevalence of hepatitis B in Asian Americans, many of them remain unscreened and untreated. A majority of the individuals chronically infected with hepatitis B virus (HBV) are not linked to care, for instance, due to a lack of culturally competent programs. There are many serious barriers preventing linkage to care (LTC), including personal, socio-cultural, and economic issues. The purpose of this study was to evaluate various barriers affecting LTC and to investigate the role and efficacy of a community-based Patient Navigator (PN) program in expediting LTC and in improving health outcomes for hepatitis B patients in a high risk population.

**Methods:**

A total of 45 individuals chronically infected with HBV were identified through community screening events and were subsequently linked to patient navigators (PN), who then arranged for the patients to have a medical evaluation with a provider of their choice in their communities. The navigators kept detailed records of the patients’ progress towards goal, and planned follow up visits for each patient. A self-report questionnaire was employed to assess patients’ demographics, history of HBV infection, and barriers in accessing health care. Specifically, the levels of importance of the barriers due to language, culture, financial reasons were assessed.

**Results:**

The study revealed that 38 of the 45 HBV infected individuals knew about their infection status from previous screening. Forty two out of 45 HBV infected individuals were linked to care within a 12 month period, demonstrating a high linkage rate. Most significant barriers identified were language and finance, followed by cultural barrier and others.

**Conclusion:**

There are specific barriers to accessing adequate care for the patients affected by chronic hepatitis B (CHB) in Korean American community. The implementation of a PN program in conjunction with the community network of health care providers may help to overcome the barriers and facilitate LTC in hepatitis B.

## Background

Chronic HBV infection causes the majority of liver related morbidity and mortality throughout the world. Up to 25 % of patients with HBV infection may suffer from liver cirrhosis, liver failure and primary liver cancer [1–3]. There is a marked disparity among racial and ethnic groups in the prevalence of chronic hepatitis B and its complications. For instance, approximately 5–10 % of all Asian Americans have HBV infection compared to 0.2 % of Caucasian Americans [4, 5]. In the United States, an estimated two million Americans are chronically infected with HBV, with a major proportion being Asian Americans. Surprisingly, a majority of these two million chronically infected people are unaware of the fact that they are infected. Furthermore, fewer than 50,000 people are currently receiving antiviral treatment in the United States, demonstrating a large disparity between the number of chronically infected individuals and the number of people receiving treatment [6, 7].

HBV screening and LTC are critical in the Asian American population for a number of reasons. With a recent immigration wave from Asia, this is the fastest growing ethnic group in the United States [8]. Although vaccination can prevent infection, rates of prevention in Asian Americans have been suboptimal [9, 10]. Secondly, not only are Asian Americans disproportionately affected by chronic hepatitis B (CHB), but they are also most likely of any population group in America to develop HBV-related liver cancer [11, 12]. Despite these grim statistics, two thirds of Asian Americans do not know they are infected, exposing a large undiagnosed population to transmission [5, 7, 10].

As hepatitis B remains significantly underdiagnosed, it is also poorly linked to adequate clinical care. The barriers to LTC in Asian Americans are multi-factorial and include personal, socio-cultural, health care system and provider-related issues. Individual obstacles mostly consist of lack of awareness about the disease, linguistic and cultural barriers, and financial issues. Additionally, providers and healthcare systems currently available in the United States lack the understanding of the significance of chronic hepatitis B. As a result, there is a serious lack of adequate health care access available for Asian Americans and other minority populations [7, 10, 13–15].

The challenges met in LTC are most often related to finding community resources, which can provide the affected population with health care services in a culturally and linguistically sensitive manner. Improvements in patient navigation, education, and counseling efforts may also foster empowerment within the community, thereby facilitating LTC from testing sites to HBV-directed medical care [7, 13, 15, 16].

This study focuses on a group of 45 Korean American individuals chronically infected with HBV from New York and New Jersey to examine various barriers in LTC. The results suggest that PN and community-based LTC not only effectively facilitate health care access, but also may address many of the health disparity issues associated with barriers in ethnic minority populations.

## Methods

### Participants

HBV infected individuals were identified from hepatitis B awareness campaigns held in northern New Jersey and metropolitan New York during the period between January 2014 and December 2014. These campaigns consisted of community-based hepatitis B screening events organized by Center for Viral Hepatitis (CVH) and Korean Community Services (KCS), which are two major non-profit Korean American organizations devoted to promoting hepatitis B screening and linkage to care. A total of 45 HBV infected individuals, who were found to have hepatitis B surface antigen, were identified. Results of the screenings were provided directly to all the individuals. Subsequently, these patients were linked to a patient navigator (PN), who then arranged for the patients to have a medical evaluation with a provider of their choice in their communities. All the HBV infected individuals who were linked to providers had a confirmatory hepatitis B DNA test. Secondly, their attendances at scheduled medical visits were individually confirmed by the PN.

### Data collection and measurement

The key variables in this study were measured by a self-report questionnaire, which consisted of three different parts. The first part of the questionnaire included demographic characteristics of gender, age, place of birth, and length of residence in the United States. The second part asked the following five questions: “When were you told you were infected with HBV?”, “Were you referred to a physician at the time of diagnosis?”, “Did you follow up with a physician after the diagnosis?”, “If not, what was the reason?”, and “Are you currently seeing a physician for hepatitis B?”. The last part of the questionnaire assessed the potential barriers of LTC. Specifically, barriers due to language, culture, and financial reasons were evaluated. In addition, structural barriers due to difficulties in obtaining transport and appointments were evaluated. Thirty six of 45 HBV infected individuals, who understood the nature of the survey, willingly participated. Using ‘Levels of Importance’ questions with scale of 0 = not at all important to 5 = most important, participants were asked to rate each barrier. The Levels of Importance were assessed for each barrier by obtaining the total number of the respondents selecting each level.

### Patient navigation program

The PN program was employed to provide HBV infected individuals with specific information on the management of CHB and to link them to providers who have expertise in hepatitis B care within their community. The PN program offered specific efforts to assist HBV infected individuals to find their way through the health care system by identifying and providing resources for Korean Americans with socioeconomic and communication barriers. There were three PNs employed in the current study. Two had BS degrees in public health, and the other PN had a BS in nursing. The PNs were trained and supervised by a group of GI and primary care physicians within the community. They were employees of community service organizations. They were not affiliated with any hospitals. Periodic quality assessments were made to address issues or problems identified through a comprehensive evaluation and to assess the stability of PN care over time.

Once all the HBV infected individuals were informed of the screening results, the PN met with them to explain the results in greater detail and to recommend a GI physician for their initial consultation. At each initial visit and follow up appointment, the PN met the patients at the providers’ office to ensure that they were guided through the process of the entire evaluation. The navigators kept detailed records of the patients’ progress towards goal, and planned follow up visits for each patient.

## Results

### Demographic characteristics of the HBV infected individuals

The sample of 45 HBV infected individuals included 24 men and 21 women whose age range was 26–70, with an average age of 46. There were 30 participants from New York and 15 from New Jersey.

### Role of patient navigation in LTC

After being informed of the results, all the HBV infected individuals were linked to PN, who then arranged for the patients to attend their medical evaluation with a provider of their choice within their communities. As a liaison between the physician and the patient, and being the advocate for the patient, the PN helped assess the patients’ condition and discuss any issues with the provider and his/her nursing staff. Navigators also played a role in educating and updating the patient and family members, if needed, to implement the care plan. This study showed that 42 out of 45 HBV infected individuals were linked to care within a 12 month period, demonstrating an overall linkage rate of 93 % (Table 1). All these 42 patients had a minimum of two visits to their providers’ offices for continuous monitoring and/or treatment. Seventeen of these patients were then started on antiviral medication.

**Table 1:** Demographic characteristics

	Number of HBV infected	Percentage (%)
Gender		
Male	24	53.3
Female	21	46.7
Total	45	100
Age		
20–29	4	8.9
30–39	8	17.8
40–49	9	20.0
50–59	15	33.3
60–69	6	13.3
70–79	3	6.7
Antiviral medication		
Yes	17	37.8
No	28	62.2
Successful LTC		
Male	22 (out of 24)	91.7
Female	20 (out of 21)	95.2

### History of HBV infection in the cohorts

We investigated how many of these HBV infected individuals might have known their infection status prior to the current screening. The survey showed that 38 (84 %) of 45 infected individuals knew about their infection status from previous screening. Only 7 (16 %) discovered that they had chronic hepatitis B from their most recent screening (Table 2).

**Table 2:** Time of diagnosis in HBV Infected Individuals

Year CHB was diagnosed	Number of HBV infected (*n* = 45)	Percentage (%)
Before 1995	13	28.9
1996–2005	14	31.1
2006–2015	18	40.0
At screening events (2014–2015)	7	15.6

Of those 38 people who were diagnosed with CHB, 36 have lived in the United States for more than 10 years. Twenty five of them were recommended to see a specialist for further evaluation. Only 4 of these infected individuals went on to see GI specialists, but did not engage in any further work up. Twenty seven (71 %) of them felt they did not seek medical care because they had no symptoms. Twenty four (63 %) said that they were not aware of potential complications of hepatitis B such as cirrhosis and liver cancer.

### Barriers in accessing health care

We also evaluated patient-related factors influencing HBV care. The survey revealed that the most significant barriers were language and finance, followed by cultural and other barriers (Table 3). If the levels of importance 3 to 5 (important to most important) are combined, cultural factors (78 %) were as significant as financial factors (72 %) in hindering the HBV infected individuals from seeking medical care. On the other hand, structural barriers due to travel and time factors were not as significant as the other barriers.

**Table 3:** Barriers in Accessing Health Care

	Level of importance (*n* = 36)					
Barriers	0*	1*	2*	3*	4*	5*
Language	0 (0 %)	1 (2.8 %)	2 (5.6 %)	5 (13.9 %)	13 (36.1 %)	15 (41.7 %)
Finance	1 (2.8 %)	4 (11.1 %)	5 (13.9 %)	6 (16.7 %)	7 (19.4 %)	13 (36.1 %)
Culture	0 (0 %)	3 (8.3 %)	5 (13.9 %)	16 (44.4 %)	7 (19.4 %)	5 (13.9 %)
Others	17 (47.2 %)	13 (36.1 %)	6 (16.7 %)	0 (0 %)	0 (0 %)	0 (0 %)

*0 = not at all important; 1–5 = increasing degree of importance

## Discussion

This study demonstrates a significant lack of CHB LTC in a high prevalence population. The results also suggest that the community-based initiatives, which mobilize local health care providers, and PN program can effectively facilitate HBV infected individuals to access and receive necessary health care services within the community.

### Lack of LTC for HBV infected individuals

A majority of Korean American HBV infected individuals identified in community-based screening events may have been previously diagnosed. As shown in Table 2, only 16 % of the HBV infected individuals identified from the current community-based screenings were newly diagnosed. In contrast, a striking 84 % had been previously diagnosed, reflecting a significant failure in LTC in this particular group. These findings are similar to the previous reports which demonstrated that only a minority of the chronically infected patients were successfully linked to care [7, 17–19]. Other investigators have also noted that up to 66 % of the HBV infected individuals screened in hospital settings were referred to care while only 40 % of those screened in community settings were referred to appropriate care [18, 19]. Although the referral percentage was greater in the group screened from the hospital setting as compared to the group screened from the community setting, there was no clear documentation on the sustainability of LTC. This issue of sustainability of LTC is critical in the care of hepatitis B since its management requires a long period of time. Furthermore, it has been frequently noted that many of those referred to the providers in a hospital setting did not continue to follow up with the providers because of various patient-related barriers.

The failure to link the HBV infected individuals to medical care has been attributed to a number of reasons. First, there are language, financial, and cultural barriers. We, therefore, conducted a survey on these HBV infected people to examine the weight of various factors prohibiting health care access in hepatitis B. The results summarized in Table 3 clearly demonstrated that language, finance, and cultural factors were all important in hindering health access in this study population. Second, patients had minimal medical knowledge about hepatitis B and its potential complications. In addition, there may be other barriers due to a lack of knowledge on the providers’ part [13].

### Community-centered approach for LTC

This study illustrates that LTC can be made successful in a community setting. The Patient Navigator program and mobilization of local health care providers who have expertise in hepatitis B care were instrumental in providing sustainable linkage from the testing site to HBV-directed care. Forty two out of 45 HBV infected individuals were successfully linked to providers within the community, revealing a high LTC rate of 93 %. PNs communicated in Korean and took individual patients through the continuum of care, ensuring that any potential barriers were adequately addressed. They also ensured that HBV infected individuals attended their first and follow up medical evaluations at their provider’s office. We learned that these patients looked for the providers, who could speak their languages, within their community. Thus, the challenging tasks in LTC may be most often related to finding qualified providers [7, 16].

There are important limitations to consider in this study. First, the participants, namely Korean American residents in New Jersey and New York, may not be representative of the overall Korean population in the United States. Education level and socioeconomic status are also important factors known to affect patients’ behavior towards health access. How these factors might have influenced the results of the current study is unknown. Secondly, the sample size of the HBV infected people is small, and many of our study subjects underwent screening knowing their hepatitis B status. For instance, some of the HBV infected participants could have utilized screening as a way of health access. Whether this would have potentially underestimated the preexisting level of LTC is a possibility. The future studies with a larger number of HBV infected individuals would be necessary to determine a more accurate level of LTC as well as to assess the sustainability of LTC in these patients.

Effective LTC with a successful implementation of PN program in the care of various diseases can enable the community to build up trust among its members, and further empower the community [20, 21]. The health status outcomes can not only help to modify individual’s health belief, but they can also influence the community’s perception of the need for health services [22]. Successful hepatitis B LTC in the community setting involves full mobilization of resources, including health care professionals who can provide culturally competent health care. Partnering with community organizations and fostering both medical and non-medical personnel are essential to promoting equitable access and improving the community’s overall health and wellbeing.

## Conclusion

There are important barriers that lead to poor linkage to care in hepatitis B for Asian Americans. The community-based patient navigation program and local health care providers can effectively engage HBV infected patients to access necessary health care services within the community.

### Ethics

All procedures performed in studies involving human participants were in accordance with the ethical standards of the institutional and/or national research committee.

## Abbreviations

CHB: chronic hepatitis B
HBV: hepatitis B Virus
LTC: linkage to care
PN: patient navigator
